# Leser-Trélat sign presenting in a patient with ovarian cancer: a case report

**DOI:** 10.4076/1752-1947-3-8583

**Published:** 2009-07-23

**Authors:** Edwin Bölke, Peter Arne Gerber, Matthias Peiper, Wolfram Trudo Knoefel, Mathias Cohnen, Christiane Matuschek, Wilfried Budach, Rainer Engers, Stephan Gripp

**Affiliations:** 1 Department of Radiation Therapy and Radiation Oncology, University of Düsseldorf, Moorenstrasse 5, 40225 Düsseldorf, Germany; 2 Department of Dermatology, University of Düsseldorf, Moorenstrasse 5, 40225 Düsseldorf, Germany; 3 Department of Surgery, University of Düsseldorf, Moorenstrasse 5, 40225 Düsseldorf, Germany; 4 Institute of Diagnostic Radiology, University of Düsseldorf, Moorenstrasse 5, 40225 Düsseldorf, Germany; 5 Department of Pathology, University of Düsseldorf, Moorenstrasse 5, 40225 Düsseldorf, Germany

## Abstract

**Introduction:**

Seborrheic keratoses are very common findings in elderly patients. However, a sudden onset and dramatic increase in the number and size of these benign lesions deserves special attention, since this may represent the Leser Trélat sign, a rare paraneoplastic cutaneous syndrome.

**Case presentation:**

A 92-year-old female presented to our clinic with multiple eruptive seborrheic keratoses, which had dramatically increased in size and number over the past two years. A diagnostic work-up revealed an ovarian carcinoma. Hence, cutaneous findings in our patient were consistent with the diagnosis of the Leser-Trélat sign.

**Conclusion:**

The Leser-Trélat sign may coincide with the diagnosis of occult cancer or follow or precede it by months or years. Practitioners should take cases of eruptive seborrheic keratoses seriously and perform thorough patient examinations.

## Introduction

Seborrheic keratoses are very common findings in elderly patients. Yet, a sudden onset and dramatic increase in the number and size of these benign lesions deserves special attention, since these may represent a rare paraneoplastic syndrome. Seborrheic keratoses may coincide with the diagnosis of occult cancer or follow or precede it by months or years. This complex is referred to as the Leser-Trélat sign.

## Case presentation

A 92-year-old woman presented to our clinic with multiple, tan to black coloured skin eruptions with rough, warty or greasy surfaces, consistent with the diagnosis of multiple seborrheic keratoses. The lesions had dramatically increased in size and numbers over the past 2 years (Panel 1 A and B). Moreover, the patient reported a 2-month history of abdominal pain and weight loss. Laboratory examinations revealed elevated CA 125 plasma levels of 500 U/ml. Clinical examination showed signs of mild ascites. Additional staging examinations, including diagnostic abdominal ultrasound and computed tomography (CT), revealed a distinctive, partly necrotic tumor (8 cm by 10 cm in diameter), originating from the left ovary and occupying the pelvis. The tumor mass was accompanied by signs of peritoneal carcinomatosis (Panel 1C). A posterior exenteration with double sided ovariectomy, hysterectomy, rectum resection and Hartmann's procedure was performed. Histopathological evaluation revealed a poorly differentiated serous papillary adenocarcinoma of the right (Panel 2A) and left (Panel 2B) ovary, infiltrating the colon, the small intestine, both Fallopian tubes and the uterus (original magnifications: A, ×40; B, ×100).

Cutaneous findings in our patient were consistent with the diagnosis of the Leser-Trélat sign. This rare paraneoplastic cutaneous syndrome is defined as the sudden onset and dramatic increase in the number and size of seborrheic keratoses as a result of cancer [1]. Skin lesions are most often observed on the back and chest, followed by the extremities, face and abdomen [1]. In almost 50 percent of affected patients the Leser-Trélat sign is accompanied by pruritus, whereas a paraneoplastic acanthosis nigricans is evident in 35 percent of the cases [bib-002 bib-003 bib-004-[4]. Predominant cancer entities in patients with the Leser-Trélat sign are gastrointestinal adenocarcinomas (stomach, liver, pancreas, colon, rectum) and lymphoproliferative disorders[1,4,5]. Moreover, the syndrome has been reported in cases of neoplasias of the breast, the lung and the urinary tract [1,6]. However, manifestations of the Leser-Trélat sign in gynaecologic malignancies and in particular in cases of ovarian cancer, as in our patient, are rare [3,7].

Pathogenetically, eruptive cutaneous paraneoplastic disorders are suspected to be induced directly by tumor-secreted growth factors [1]. Accordingly, various authors have described an association of the Leser-Trélat sign with increased levels of transforming growth factor alpha (TGF-α), insulin-like growth factor, epidermal growth factor (EGF) or an increased expression or activity of the epidermal growth factor receptor (EGFR) [1,8]-[10]

## Conclusion

Whereas seborrheic keratoses are very common findings in elderly patients, the eruptive appearance of a number of these benign skin tumors requires further examination. Since these lesions may coincide with the diagnosis of occult cancer, or follow or precede it by months or years, practitioners should perform a thorough examination including taking a complete patient history, physical examination, routine blood tests, chest radiography, abdominal ultrasound or computed tomography (CT), as well as mammography and a Papanicolaou smear test in women and prostate-specific antigen testing in men. With regard to the high incidence of gastrointestinal neoplasias, additional endoscopic analyses (esophagogastroduodenoscopy and colonoscopy) should be considered [4].

In our case the patient was treated with surgery and at the time of writing is receiving palliative chemotherapy with carboplatin.

**Figure 1:**
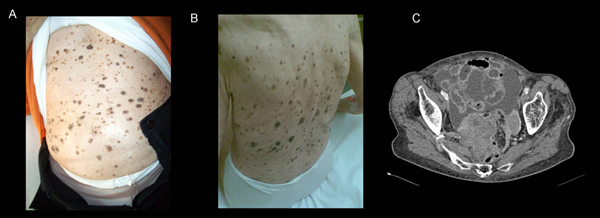
****(A)** and **(B)** Widespread seborrheic keratoses**. **(C)** Computed tomography scan showing a necrotic tumor accompanied by signs of peritoneal carcinomatosis.

**Figure 2:**
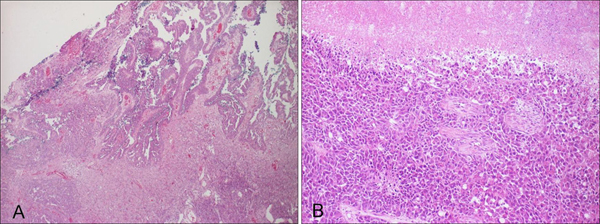
**Histopathological evaluation revealing a poorly differentiated serous papillary adenocarcinoma of the right **(A)** and left **(B)** ovary, infiltrating the colon**. Original magnifications: A, ×40; B, ×100.

## Authors' contributions

EB and PAG analyzed and interpreted the patient data. RE performed the histological examination. All authors read and approved the final manuscript.

## Consent

Written informed consent was obtained from the patient for publication of this case report and accompanying images. A copy of the written consent is available for review by the Editor-in-Chief of this journal.

## Competing interests

The authors declare that they have no competing interests.
